# Delirium in the context of the other brain reaction types

**DOI:** 10.1093/ajrccm/aamag124

**Published:** 2026-03-21

**Authors:** Jan N M Schieveld, Jacqueline J M H Strik, the other members of the MUMC (Maastricht University Medical Centre) Study Group on Delirium, Jan N M Schieveld, Jacqueline J M H Strik

**Affiliations:** Department of Psychiatry and Psychology, Division of Child and Adolescent Psychiatry and Psychology, Maastricht University Medical Centre+, Maastricht, the Netherlands; School for Mental Health and Neuroscience (MHeNs), Maastricht University, Maastricht, the Netherlands; School for Mental Health and Neuroscience (MHeNs), Maastricht University, Maastricht, the Netherlands

In critical illness, the brain always is involved as well. Due to (critical) illness, the body and the brain can react with many signs and symptoms of (imminent) dysregulation. The 2 most important sets of these are vital signs and brain reaction types.

The classic vital signs are level of consciousness, respiratory pattern and rate, blood pressure, pulse pattern and rate, and body temperature. These 5 signs are well known, and the answer to the question, “Any delirium: Yes or No?” progressively is considered to be the sixth.^1^ According to the *Diagnostic and Statistical Manual of Mental Disorders* (*DSM-5*), the 2 core diagnostic criteria for delirium are (1) a disturbance of attention and awareness and (2) of neurocognition. However we argue that these are highly common and nonspecific aspects of the executive (dis)functioning, and these must lead to many false-positive delirium diagnosis (please see below in the text). The brain also can react with a number of other common, but not very well known, neuropsychiatric–neuropsychological clinical syndromes. The 5 basic ones are sickness behavior, executive dysfunction, formal thought disorders, sleep–wake cycle disturbance, and apathy. Each of these syndromes has a unique clinical signature. Sickness behavior features social withdrawal, emotional lability, and loss of appetite. Executive dysfunction, appearing from the 1970s in the literature, impairs attention (“first out and last back”), working memory, and cognitive control, which are core aspects of decision making and self-regulation.^2^ Formal thought disorder presents as either highly disorganized (positive) or slowed and minimal (negative) speech and thinking.^3^ Disturbance in sleep wake cycle occurs frequently in neuropsychiatric dysfunction and can often be the first sign of brain reaction in somatic illness. Apathy syndrome involves deficits in affect, behavior, and cognition—the ABCs of motivation—leading to a lack of initiative or response to stimuli. Apathy is highly prevalent in combination with already existing neuropsychiatric disorders in noncritically ill patients, and so it can be already preexisting.^4^

Other neuropsychiatric reaction types can occur as well: psychosis, epilepsy, and catatonia. These 3 disorders can be finally complicated by a disturbance of arousal and can rapidly shift and or coexist, confounding clinicians and researchers alike. And so these remain misunderstood and neglected and are hardly integrated into the current understanding of delirium.

Figure 1 presents now—and to our knowledge for the first time—clearly and coherently and in a logically ordered and gradual way, these 9 common reaction types of the brain in the context of (critical) illness. This flowchart reads from top (the onset of critical illness) to bottom (disturbance of arousal).

**Figure 1:**
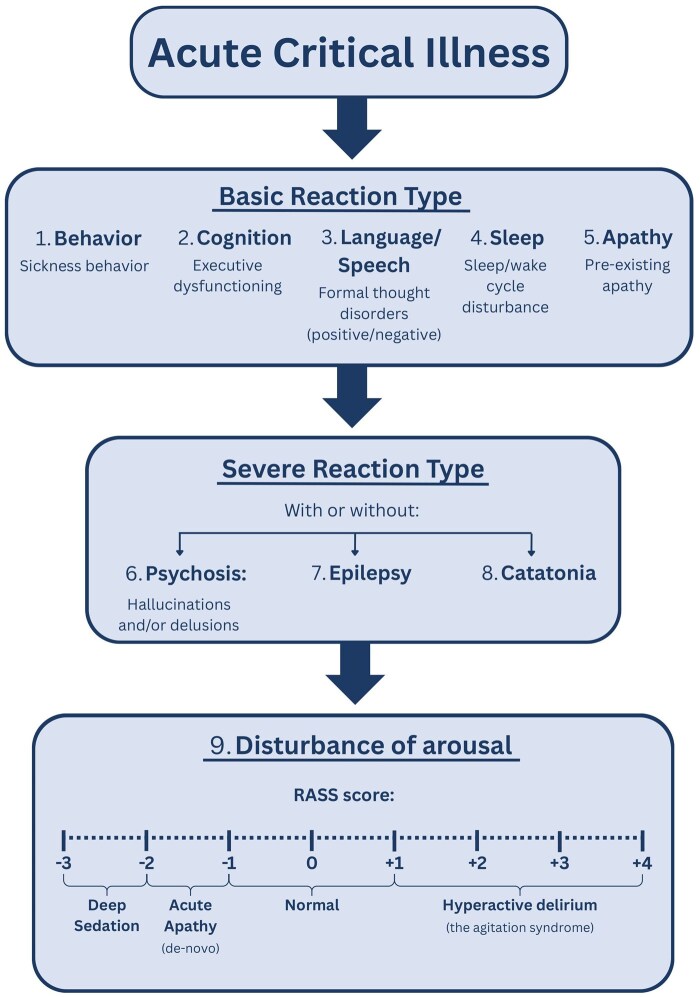
Flowchart of the nine common neuropsychiatric and neuropsychological brain reaction types due to (critical) illness.

The disturbances in the domains—behavior, cognition (executive dysfunctioning), language/speech (formal thought disorders) and sleep–wake cycle—already are common in family medicine/general practice. They frequently occur in, for example, serious influenza, COVID-19, Alzheimer disease, or during the onset of menopause. And so we argue that these are not essential core criteria. Psychosis, epilepsy, and catatonia are less prevalent but more serious and require urgent specialized medical attention. With the awareness of the existence of these reaction types, so in this broader context, one assesses the level of arousal. Because of the important clinical fact that apathy and agitation are opposite poles within the same overarching arousal spectrum, they are presented and discussed at last.

By doing so we created in one glance the most natural and complete overview, resulting in a flowchart, which is presented in Figure 1. It clearly illustrates that delirium exists in the context of these other brain reaction types and that it must be evaluated accordingly. It also shows serendipitously that it must be finally understood as a disturbance of arousal and not primarily as a disturbance of executive function. After all, just as a cough is not pneumonia per se, so is a disturbance of executive function not delirium per se. So the agitation syndrome is hyperactive delirium, and the inhibition syndrome, in the Richmond Agitation–Sedation Scale window of -1 to -2, is hypoactive delirium/acute apathy syndrome de novo. These 2 opposite disorders of arousal are not the same delirium phenotype, but 2 totally different ones. This is another major cause of false-positive delirium diagnosis. Acute apathy syndrome de novo differs significantly regarding arousal level (inhibition), epidemiology (much higher prevalence), outcome (worse), and antipsychotic treatment response: none.^5^ Multidisciplinary awareness across and outside the medical domain is urgently needed. This delirium issue is highly prevalent especially in critical care, and in geriatric medicine. Given the rapidly growing emancipation and empowerment of patients and their families, an integrated delirium understanding and awareness are also relevant not only for the general public but also for administrators, the government, and medical insurance companies, and so there also exists a huge and important public health interest.

We finally propose logical adaptations in the next editions of the *International Classification of Diseases* and of the *DSM* (the scientific and clinical contexts) as well as refinements to the interpretation of the *D* component of the ABCDEF bundle.^6^ Specifically, we suggest (1) incorporation of sickness behavior, executive functioning, formal thought disorders, sleep–wake cycle problems, and the apathy syndrome, together with psychosis, epilepsy, and catatonia, into the broader consideration of the delirium context and of its characteristic diagnostic criteria; and (2) encouraging routine specification of delirium phenotypes, particularly along the arousal spectrum. Only so we can do justice to all 9 specific brain reaction types and can we make the next nuanced step forward to take care of the endangered brain. Only so we can enhance precision medicine, and then we are able to develop more tailored interventions regarding prevention and treatment, to improve outcomes for our patients and families.

## Supplementary Material

aamag124_Supplementary_Data
